# Stomatal Control and Leaf Thermal and Hydraulic Capacitances under Rapid Environmental Fluctuations

**DOI:** 10.1371/journal.pone.0054231

**Published:** 2013-01-24

**Authors:** Stanislaus J. Schymanski, Dani Or, Maciej Zwieniecki

**Affiliations:** 1 Department of Environmental Systems Sciences, ETH Zurich, Zurich, Switzerland; 2 Department of Plant Sciences, University of California Davis, Davis, California, United States of America; Colorado State University, United States of America

## Abstract

Leaves within a canopy may experience rapid and extreme fluctuations in ambient conditions. A shaded leaf, for example, may become exposed to an order of magnitude increase in solar radiation within a few seconds, due to sunflecks or canopy motions. Considering typical time scales for stomatal adjustments, (2 to 60 minutes), the gap between these two time scales raised the question whether leaves rely on their hydraulic and thermal capacitances for passive protection from hydraulic failure or over-heating until stomata have adjusted. We employed a physically based model to systematically study effects of short-term fluctuations in irradiance on leaf temperatures and transpiration rates. Considering typical amplitudes and time scales of such fluctuations, the importance of leaf heat and water capacities for avoiding damaging leaf temperatures and hydraulic failure were investigated. The results suggest that common leaf heat capacities are not sufficient to protect a non-transpiring leaf from over-heating during sunflecks of several minutes duration whereas transpirative cooling provides effective protection. A comparison of the simulated time scales for heat damage in the absence of evaporative cooling with observed stomatal response times suggested that stomata must be already open before arrival of a sunfleck to avoid over-heating to critical leaf temperatures. This is consistent with measured stomatal conductances in shaded leaves and has implications for water use efficiency of deep canopy leaves and vulnerability to heat damage during drought. Our results also suggest that typical leaf water contents could sustain several minutes of evaporative cooling during a sunfleck without increasing the xylem water supply and thus risking embolism. We thus submit that shaded leaves rely on hydraulic capacitance and evaporative cooling to avoid over-heating and hydraulic failure during exposure to typical sunflecks, whereas thermal capacitance provides limited protection for very short sunflecks (tens of seconds).

## Introduction

Leaves may be subjected to rapidly fluctuating irradiance due to motion of sunflecks and clouds that may span two orders of magnitude from light compensation points of shade-adapted leaves to almost full irradiance intensities [1]. Such environmental fluctuations occur at time scales (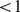 min) much shorter than characteristic time scales for stomatal adjustments (2 to 60 min.) [2]. For leaves with slowly adjusting stomata, rapid fluctuations at shorter time scales could push leaf hydraulic and thermal status beyond operational limits resulting in xylem cavitation, overheating or wilting. Chazdon [1] pointed out that whereas intense sunflecks may lead to an increase in leaf temperatures by 18 K, heat damage due to such occurrences was rarely observed. Thenceforth, most analyses of stomatal adjustments to fluctuating irradiance in the canopy tended to focus on carbon gain and water stress, and much less on the need to avoid heat damage (e.g. [1]–[5]). On the other hand, Beerling et al. [6] simulated steady-state leaf temperatures of planar leaves with low and high stomatal numbers and concluded that high stomatal density is necessary to allow for sufficient evaporative cooling and avoid lethal leaf temperatures (assumed in the range of 45–55°C) under high irradiance.

Since evaporative cooling is essential to avoid heat damage in leaves exposed to full sunlight, and time scales of stomatal adjustments are longer than fluctuations in solar irradiance within a canopy, the question arises whether typical sunfleck intensities and durations could damage non-transpiring leaves. If this is the case, then adaptation for cooling would appear as a more imperative driver for stomatal adjustments than the potential increase in carbon gain, assumed in most studies on sunfleck effects to date.

The interlinked leaf thermal and hydraulic capacitances (embedded in leaf water content per leaf area) may provide passive protection and thus play a critical role in autonomous capacitive-based responses to rapid fluctuations in irradiance. For example, a variable leaf water content per unit leaf area can affect both thermal and hydraulic capacitances. When a leaf is exposed to a sunfleck, its temperature can rise by up to 20 K with an initial rate of 1–2 K min^−1^ for leaves with about 50–100 g m^−2^ water content [7]. Given the effect of leaf temperature on leaf-to-air vapour pressure gradient, transpiration rates are expected to rise accordingly. Increasing leaf water content (thicker leaves) can be an effective measure to increase capacitive buffering of such environmental fluctuations, until more robust but slower regulatory measures such as stomatal adjustments can take over and prevent detrimental effects.

An alternative protective measure may involve keeping stomata open even under low light conditions, in anticipation of autonomous evaporative cooling in response to a rapid increase in irradiation. The necessity to avoid damaging temperatures may thus impact water use efficiency in water-limited environments. Researchers have found that a number of shade tolerant species maintain open stomata and very low water use efficiencies in the shade, while others maintain lower stomatal conductances in the shade but are able to open their stomata faster in response to a sunfleck (e.g. [1], [3], [8]).

An important factor to consider is that a spike in transpiration flux due to rapidly changing environmental conditions (e.g. due to a sunfleck or wind gust), may trigger cavitation and failure of the water supply network to the leaf [9]. To mitigate such a scenario, stored water in leaf tissue could buffer the effect of such a spike in demand and thus reduce the risk of cavitation. For a range of living plant tissues including leaves, the water content can vary by up to 10% of its maximum value before turgor loss and irreversible plasmolysis sets in [10]–[14]. Consequently a leaf with a water storage of 0.2 mm (0.2 kg m^−2^) could lose up to 0.02 mm of water (0.02 kg m^−2^) before permanent damage occurs. In this context, turgor loss and passive stomatal closure can be seen as an autonomous measure to stop water loss before this critical stage is reached. Furthermore, it has been shown for a number of tree species that leaves are more vulnerable to xylem embolism than stems [15]–[18], suggesting that the hydraulic pathways in trees are organised in a way to protect the stem xylem from pressure drops emanating from the leaves [18].

Considering the disparity in time scale of environmental fluctuations relative to stomatal adjustment times, the primary objective of this study is to investigate the protective roles of leaf heat and water capacitances under fast environmental fluctuations (relative to stomatal response times).

We aim to answer the following questions:

- Do natural fluctuations in leaf irradiance necessitate stomatal regulation to avoid heat damage or hydraulic failure?
- What is the role of leaf heat and water capacities in negotiating the trade-off between cavitation and over-heating?

A physically-based leaf energy balance model was formulated to simulate leaf temperature and transpiration dynamics as a function of varying environmental conditions (irradiance, air temperature, vapour pressure, wind speed). The effect of rapid environmental fluctuations (e.g. irradiance due to moving sunflecks) on the heat and mass exchange of the leaf and resulting changes in leaf temperature and hydration status were simulated. In a first step, simulations were performed using an observed time series of irradiance and air temperatures in the understorey of a tropical rainforest [19], which allowed comparison of simulated leaf temperature dynamics with observations. In a second step, typical amplitudes and time scales of irradiance fluctuations were considered to investigate the importance of leaf heat and water capacities for avoiding damaging extremes in leaf temperatures and hydration status.

## Methods

All relevant symbols used in this section and their respective units are given in Table 1. All derivations and analyses were performed using the freely available software SAGE (version 5.0, http://sagemath.org). The steady-state temperature for given leaf dimensions, environmental conditions and stomatal conductance (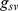) was obtained by numerical root finding of Eq. 1 (see below), whereas the dynamics were simulated using a finite time step discretisation.

**Table 1:** Symbols, standard values and units used in this paper.

Symbol	Description (standard value)	Units
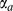	Thermal diffusivity of air	m^2^ s^−1^
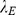	Latent heat of vaporisation ( 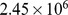 )	J kg^−1^
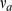	Kinematic viscosity of air	m^2^ s^−1^
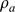	Density of dry air	kg m^−3^
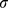	Stefan-Boltzmann constant ( 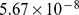 )	W m^−2^ K^−4^
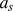	Fraction of transpiring leaf surface area (relative to 1-sided leaf area)	-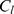	Conductive heat flux away from leaf subsection	W m^−2^
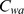	Concentration of water vapour in the free air	mol m^−3^
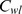	Concentration of water vapour inside the leaf	mol m^−3^
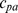	Specific heat of dry air (1010)	J K^−1^ kg^−1^
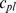	Leaf heat capacity at constant pressure	J K^−1^ m^−2^
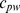	Heat capacity of water at constant pressure	J K^−1^ kg^−1^
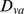	Binary diffusion coefficient of water vapour in air	m^2^ s^−1^
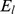	Latent heat flux away from leaf	W m^−2^
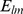	Transpiration rate	kg m^−2^ s^−1^
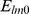	Steady-state transpiration rate prior to arrival of sunfleck	kg m^−2^ s^−1^
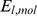	Transpiration rate in molar units	mol m^−2^ s^−1^
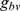	Leaf boundary layer conductance to water vapour	m s^−1^
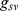	Stomatal conductance to water vapour	m s^−1^
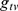	Total leaf conductance to water vapour	m s^−1^
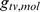	Total leaf conductance to water vapour	mol m^−2^ s^−1^
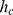	Average one-sided convective heat transport coefficient	m s^−1^
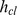	Convective heat transport coefficient for the lower leaf side	W K ^−1^ m^−2^
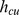	Convective heat transport coefficient for the upper leaf side	W K ^−1^ m^−2^
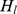	Sensible heat flux emitted by the leaf	W m^−2^
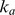	Thermal conductivity of air in leaf boundary layer	W K^−1^ m^−1^
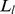	Characteristic leaf length scale (0.05)	m
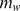	Leaf water content	kg m^−2^
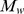	Molar mass of water (0.018)	kg mol^−1^
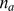	Amount of matter	mol
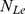	Lewis number	-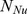	Nusselt number	-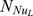	Average Nusselt number for whole leaf	-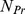	Prandtl number for air (0.71)	-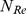	Reynolds number	-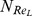	Average Reynolds number for whole leaf	-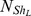	Average Sherwood number	-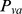	Vapour pressure in free air	Pa
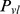	Vapour pressure inside the leaf	Pa
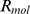	Molar gas constant (8.314472)	J K^−1^ mol^−1^
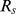	Absorbed short wave radiation	W m^−2^
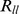	Net longwave radiation emission by a leaf	W m^−2^
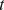	Time	s
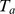	Air temperature	K
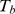	Boundary layer temperature, 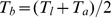	K
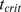	Critical time to heat damage or turgor loss	s
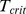	Critical leaf temperature for the onset of heat damage (322)	K
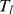	Leaf temperature	K
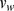	Wind velocity	m s^−1^
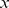	Distance from leading edge along a leaf	m

All area-related variables are expressed per unit leaf area.

### Leaf energy balance model

**Figure 1:**
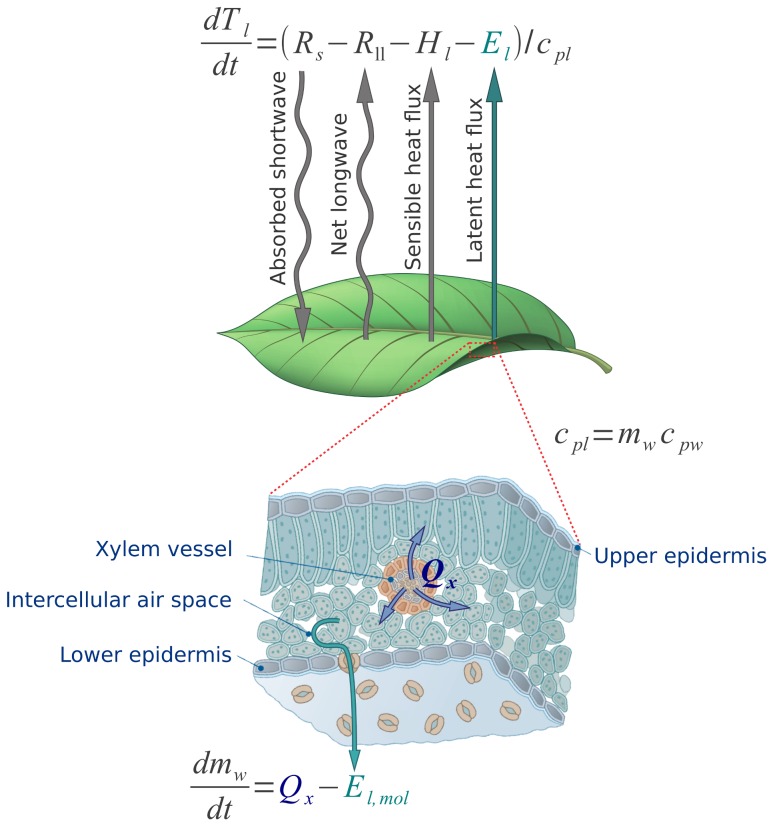
Components of the leaf mass and energy balance and their conventional directions considered in this study. Arrows point in the direction of a positive flux. Both leaf temperature (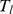) and water content (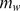) depend on the transpiration rate (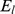 and 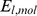 in energetic and molar units respectively). The leaf water content (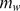) affects the leaf heat capacity (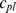) and turgor pressure, which becomes critical when leaf water content declines below 90% of its maximum value (see text). Changes in leaf water content result from differences in the water supply rate from the xylem (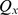) and evaporative losses (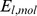).

The leaf energy balance is determined by the dominant energy fluxes between the leaf and its surroundings, including radiative, sensible, and latent energy exchange (linked to mass exchange). The dominant energy fluxes considered here are illustrated in Fig. 1.

Neglecting heat transport through the petiole, the energy balace of a spatially homogeneous leaf can be written as:
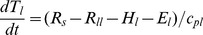
(1)where 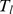 is leaf temperature, 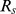 absorbed short wave radiation, 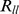 is the net longwave balance, i.e. the emitted minus the absorbed, 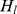 is the sensible heat flux away from the leaf, 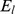 is the latent heat flux away from the leaf and 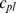 is the leaf heat capacity at constant pressure. In the above, extensive variables are defined per unit leaf area.

The special case of a partly illuminated leaf would involve formulation of the energy balances for the illuminated and the shaded leaf areas separately and an additional term for the heat transport by conduction between these two leaf subsections (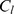):
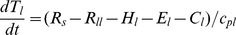
(2)where all terms refer to the sunlit part of the leaf. For simplicity, we will limit the present analysis to spatially homogeneous planar leaves, i.e. full illumination and a negligible temperature gradient between the two sides of the leaf.

Assuming that leaf heat capacity is mainly determined by its water content (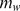), 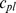 is represented as:
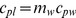
(3)where 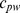 is the heat capacity at constant pressure of liquid water.

Assuming further that the longwave radiation absorbed by the leaf is equal to its emission at air temperature (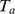), the net longwave emission is represented by the difference between blackbody radiation at 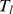 and that at 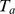:
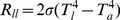
(4)where 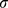 is the Stefan-Boltzmann constant and the factor 2 represents the two sides of a broad leaf. Note that this formulation is a coarse approximation, but it represents a standard procedure (e.g. [20]). A more accurate account of the longwave radiation balance would have to involve longwave sky radiation as well as longwave radiation originating from the ground and neighbouring leaves in the canopy.

#### Sensible heat flux

The exchange of sensible and latent heat between the leaf and the free air is dominated by convective transport, which is generally formulated as the product of a convective transport coefficient and the temperature difference between the surface and the free air. Convective transport coefficients depend on leaf orientation, geometry, and surface properties (e.g. hairs), wind conditions and temperature (p. 168–172 in [20]). In this study, we neglect the effects of leaf surface properties, orientation and geometry by assuming that leaves behave like horizontal rectangular metal plates of width 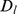 (in wind direction).

The total convective heat transport away from the leaf is represented as:
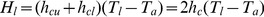
(5)where 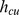, 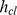 and 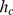 are the convective heat transport coefficients for the upper, the lower and the average of both leaf sides respectively.

Different textbooks propose different empirical equations to calculate heat transfer coefficients for flat plates. The differences may originate from different experimental data, different reference length scales or different boundary conditions. In order to avoid the risk of mismatch between empirical equations and applicable boundary conditions and for better traceability, we drew most of the below relations from a single textbook (Incropera et al., 2006 [21]).

Following Incropera et al. [21], different convective heat transfer coefficients were formulated for forced and free convection (presence and absence of significant wind), and laminar vs. turbulent conditions. The coefficients are generally formulated as a function of the dimensionless Nusselt number (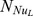):
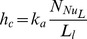
(6)where 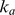 is the thermal conductivity of the air in the boundary layer and 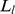 is a characteristic length scale of the leaf. In the absence of wind, buoyancy forces, driven by the density gradient between the air at the surface of the leaf and the free air dominate convective heat exchange (free or natural convection). The influence of vapour pressure gradients across the stomatal pores on the density gradient would add a significant level of complexity to the solution of the sensible and latent heat exchange equations. For simplicity, we will therefore limit this study to forced conditions, i.e. where wind velocity is greater than 0.5 m s^−2^ for the leaf properties and environmental conditions considered here.

Under strong enough wind, inertial forces drive the convective heat transport (forced convection) and the relevant dimensionless number is the Reynolds number (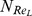), which defines the balance between inertial and viscous forces:
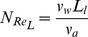
(7)where 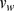 is the wind velocity (m s^−1^), 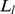 (m) the length of the leaf in wind direction and 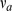 is the kinematic viscosity of air.

The local Reynolds number changes from the leading edge downwind as ([21], Eq. 6.23):
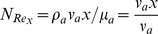
(8)where 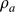 is the air density, 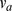 is the wind velocity outside the boundary layer, 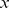 is the distance from the leading edge, 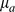 is the dynamic viscosity and 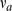 is the kinematic viscosity of air (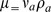). All of the fluid properties are evaluated at the mean boundary layer temperature, defined as 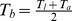 ([21], Eq. 7.2).

Integrated over the whole leaf, the average Reynolds number (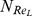) is given by Eq. 7. For an isothermal flat plate with a fully laminar boundary layer, the average Nusselt number is given as ([21], Eq. 6.23):
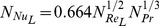
(9)where 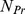 is the dimensionless Prandtl number (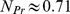 for air).

At a certain distance from the leading edge, 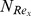 can reach a critical number (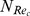) and flow transitions from laminar to turbulent flow. This critical Reynolds number depends on the surface roughness and the turbulence level of the free stream but is known to vary from about 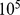 to 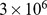 ([21], P. 361). As opposed to purely laminar flow, where 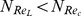, cases where a part of the boundary layer is turbulent (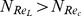) are referred to as mixed flow. Since turbulent convection is stronger than laminar convection, lower values of 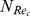, implying earlier transition to turbulent flow, would lead to enhanced sensible heat flux. For mixed flow over an isothermal plate (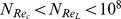), Incropera et al. gives the following empirical formulation ([21], Eq. 7.38):
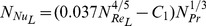
(10)with
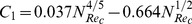
(11)


Incropera et al. ([21], P. 412) states that 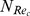 can be as low as 0 if the flow is “tripped” at the leading edge of the object using some mechanical turbulence promotor. However, we found that the equation does not give reasonable results for 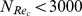, as the resulting Nusselt number would be lower than that for fully laminar flow (Eq. 9). Eq. 10 is identical with Eq. 9 if 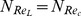. Thus, to make it valid across the whole range, we modified 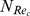 such that it takes values of 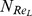 if 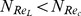. This was achieved by substituting 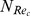 by the term 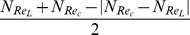 in Eq. 11.

It is interesting to note that experiments with real leaves revealed an enhanced forced convection by a factor of up to 2.5 compared to flat plates of similar dimensions in laminar flow [22], [23]. This was largely attributed to the level of turbulence already present in canopy wind. However, this does not seem to be consistent with the variation of critical Reynolds numbers attributed to the level of turbulence by Incropera et al., which was estimated to be in the range of 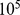 to 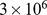 ([21], P. 361). Within this range, a leaf of 5 cm width would only start experiencing turbulence at wind velocities of above 31 m s^−1^ (Eq. 8). Even the lowest critical Reynolds number of 3000, for which Eq. 10 is still applicable, would only lead to an onset of turbulence at wind velocities of above 1 m s^−1^, which is still above the maximum wind velocity of 0.4 m s^−1^ used in the experiment by Parlange and Waggoner [24], so the observed enhancement in sensible heat flux cannot be simulated using the formulations given above. To get as close as possible to real leaves while using the established relationships for heated plates, we used a critical Reynolds number of 3000 rather than the 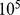 suggested by Incropera et al. [21].

#### Latent heat flux

Evaporation from a wet leaf was formulated as a function of the concentration of water vapour inside the leaf (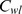, mol m^−3^) and in the free air (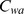, mol m^−3^) ([21], Eq. 6.8):
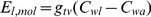
(12)where 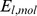 (mol m^−2^ s^−1^) stands for a flux of matter and 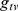 (m s^−1^) is the total conductance for water vapour.

For transpiration through stomata, 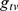 is the combination of boundary layer and stomatal conductances (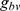 and 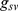 respectively), derived from the assumption that stomatal and boundary layer resistances are in series and using the definition of conductances as the inverse of resistances:
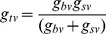
(13)


The concentration difference in Eq. 12 is a function of the temperature and the vapour pressure differences between the leaf and the free air. Assuming that water vapour behaves like an ideal gas, we can express its concentration as:
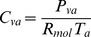
(14)where 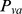 is the vapour pressure, 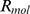 is the universal gas constant and 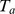 is the temperature. In this study the vapour pressure inside the leaf is assumed to be the saturation vapour pressure at leaf temperature, which is computed using the Clausius-Clapeyron relation (Eq. B.3 in [25]):
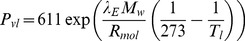
(15)where 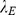 is the latent heat of vaporisation and 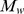 is the molar mass of water. The conversion of the vapour flux in molar units to latent heat flux in energetic units was done by multiplying 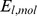 by the molar mass of water and the latent heat of vaporisation:
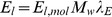
(16)


Note that 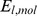 is commonly expressed as a function of the vapour pressure difference between the free air (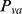) and the leaf (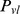), in which the conductance (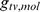) is expressed in molar units (mol m^−2^ s^−1^):
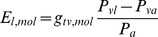
(17)For 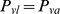, Eq. 12 can still give a flux, whereas Eq. 17 gives zero flux. This is because the concentrations of vapour in air (mol m^−3^) can differ due to differences in temperature, even if the partial vapour pressures are the same (see Eq. 14). Therefore, the relation between 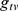 and 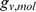 has an asymptote at the equivalent temperature. It can be obtained by combining Eqs. 12 and 17 and solving for 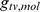:
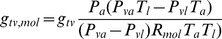
(18)For 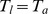, the relation simplifies to:
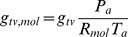
(19)which, for typical values of 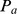 and 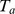 amounts to 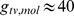 mol m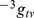. For all practical purposes, we found that Eqs. 12 and 17 with 
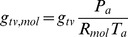
 give similar results when plotted as functions of leaf temperature.

#### Boundary layer conductance to water vapour

The boundary layer conductance in Eq. 13 is equivalent to the mass transfer coefficient for a wet surface ([21], Eq. 7.41):
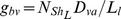
(20)where 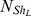 is the dimensionless Sherwood number and 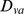 is the diffusivity of water vapour in air. If the convection coefficient for heat is known, the one for mass (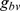) can readily be calculated from the relation ([21], Eq. 6.60):
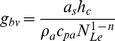
(21)where 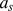 is the fraction of one-sided transpiring surface area in relation to the surface area for sensible heat exchange, 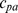 is the constant-pressure heat capacity of air, 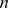 is an empirical constant (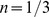 for general purposes) and 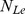 is the dimensionless Lewis number, defined as ([21], Eq. 6.57):
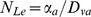
(22)where 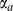 is the thermal diffusivity of air. The value of 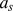 was set to 0.5 for leaves with stomata on one side only, and to 1.0 for stomata on both sides. Other values could be used for leaves only partly covered by stomata.

#### Model closure

Progressively inserting Equations 14, 15, 13, 21, 6, 22, 10 (or 9) and 7 into Equation 12 gives an expression for the transpiration flux as a function of leaf temperature, where we still need to calculate 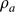, 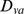, 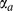, 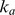, and 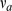, while 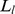, 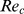 and 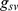 are prescribable leaf properties, and 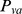 and 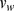 (vapour pressure and wind speed) are part of the environmental forcing. 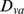, 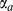, 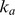 and 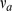 were parameterised as functions of boundary layer temperature only, by fitting linear curves to published data ([20], Table A.3):
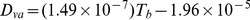
(23)

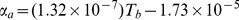
(24)

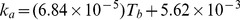
(25)

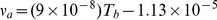
(26)Assuming that air and water vapour behave like an ideal gas, and that dry air is composed of 79% N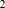 and 21% O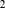, we calculated the density as a function of temperature, vapour pressure and the partial pressures of the other two components using the ideal gas law:
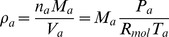
(27)where 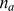 is the amount of matter (mol), 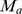 is the molar mass (kg mol^−1^), 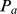 the pressure, 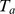 the temperature and 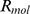 the molar universal gas constant. This equation was used for each component, i.e. water vapour, N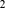 and O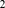, where the partial pressures of N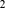 and O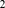 are calculated from atmospheric pressure minus vapour pressure, yielding:
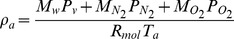
(28)where 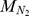 and 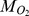 are the molar masses of nitrogen and oxygen respectively, while 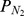 and 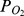 are their partial pressures, calculated as:
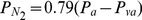
(29)and
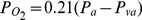
(30)


### Simulation of observed leaf temperature dynamics

To test whether the leaf energy balance model produces reasonable results and how leaf heat capacity could affect leaf temperature dynamics in a natural environment, we simulated the dynamics of leaf temperature of *Shorea leprosula* seedlings in response to observed fluctuations in solar irradiance in a rainforest understory and compared the results with observed leaf temperature fluctuations [19].

**Figure 2:**
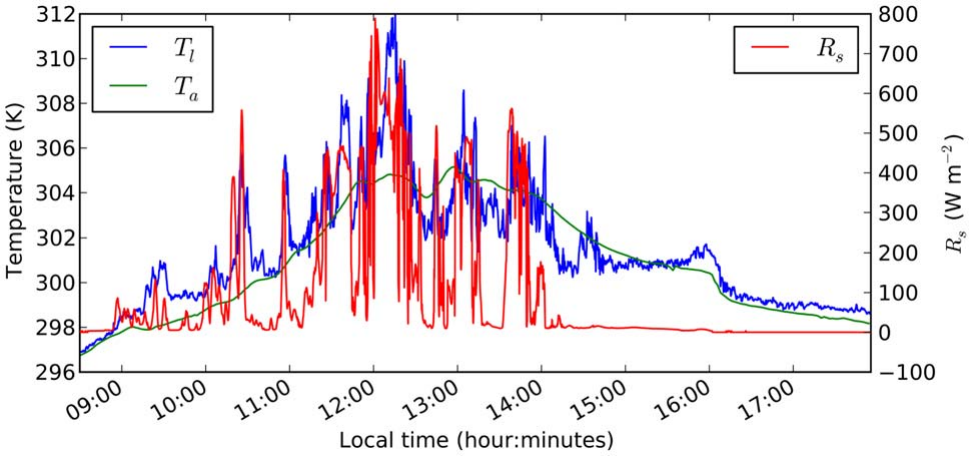
Observed irradiance (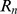), air temperature (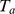) and leaf temperature (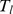) in the understorey of a tropical rainforest. Data converted from [19].

The forcing data set consisted of air temperature measured in two minute intervals and solar radiation measured in 10 second intervals. The observed leaf temperatures were also reported in 10 second intervals, all for a single day from 8:30am to 6pm (Fig. 1 in [19]). Andrew Leakey kindly provided the original data for the analysis. To convert photosynthetically active photon flux density (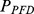, 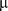
mol m^−2^ s^−1^) recorded by the quantum sensor SKP 215 (Skye Instruments) to shortwave irradiance (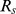, W m^−2^), we used a conversion coefficient of 4.57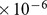 mol J^−1^ [26]. Then we expanded from the photosynthetically active range of 400–700 nm to the full shortwave range of 200–4000 nm by using a conversion coefficient of 0.45, which was derived from an online database [27]: 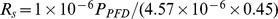. Air temperature was linearly interpolated to obtain values at the same time steps as 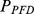. The resulting data set is shown in Fig. 2.

The leaves of the *Shorea leprosula* seedlings had dimensions of approximately 130
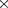
45 mm and a specific leaf area of 19 mm^2^ mg^−1^ dry matter (pers. comm. Andrew Leakey). Leaf thickness of *Shorea leprosula* in the understorey was reported in the range of 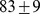 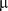
m (P. 370 in [28]). Assuming a 1∶1 partitioning between leaf dry matter and water content, we found that a water content of 0.05 kg m^−2^ would be reasonable, leaving 0.03 mm of the leaf thickness for dry matter and air. Any higher water content would have to result in greater leaf thickness. As a consequence we used 0.05 m as the characteristic length scale of the leaf and a heat capacity equivalent to a leaf water content (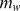) of 0.05 kg m^−2^ for the simulations.

### Reference threshold for time to heat damage

Exposure of living plant tissue to excessive heat can cause immediate (direct) or delayed (indirect) damage. Heat damage not only depends on exposure temperature, but also on the duration of the exposure. Heat vulnerability can vary between species, and also over time, due to acclimation and so-called hardening in response to prior non-lethal exposures to high temperatures [29], [30]. In order to establish a realistic reference for heat damage as a result of dynamic exposure to high leaf temperatures, we used results obtained from experiments on black spruce (*Picea mariana*) twigs, performed by Colombo & Timmer [30]. It is not the purpose of this study to assess the heat vulnerability of a particular or representative species; we just use this one example as a reference for assessing potential heat damage risks related to rapid and short-lived leaf temperature rises due to sunflecks.

**Figure 3:**
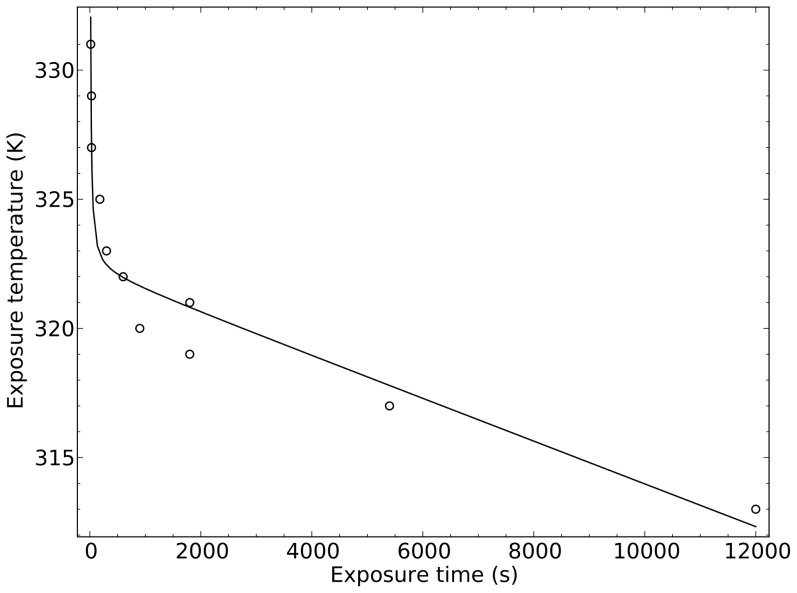
Fit of Eq. 31 to data in [30, Tab. 2]. 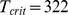
 K, 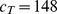 K s, and 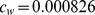 K s^−1^, standard root mean square deviation: 0.07.

Colombo et al. [30] conducted extensive heat exposure experiments on black spruce needles and found that the critical exposure time and temperature are related exponentially. In the experiments, spruce twigs were submerged in water of varying temperatures for varying time periods and the percentage of damage was recorded. We have to consider that the submersion itself had a damaging effect in addition to the heat, as it is clear that a twig submerged for long enough would get damaged no matter what the temperature is. To separate these effects, we used the following formulation for the critical exposure temperature (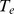) as a function of submersion time (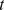):
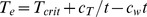
(31)where 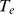 (K) is the exposure temperature, 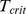 (K) is the critical temperature below which no damage occurs, 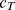 (K s) is a constant determining the effect of exposure time (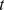, s) and 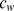 (K s^1^) is a constant representing the effect of the submersion alone. A least-square fit of this model to the exposure temperature and duration data presented by Colombo et al. ([30], Tab. 2) revealed 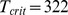 K, 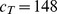 K s, and 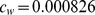 K s^−1^ (Fig. 3). This suggests that the critical temperature for heat damage is around 49°C and the damaging time amounts to 148 seconds per Kelvin above that threshold, i.e. damage happens when 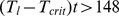 K s. It further suggests that the submersion effect lowers the recorded damaging temperature by 0.0008 K per second of submersion time. Using these values as a reference, we computed the critical time (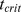 to heat damage as the time when the integral of 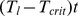 reaches 148 Ks, starting when 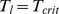.

**Table 2:** Natural and experimental light fluctuations vs. stomatal conductances.

Obs. 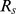	Obs. 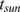	Exp. 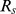	Min. 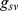	Max. 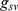	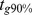	Reference
50–750	300–1200	150–850	0.0047	0.01	300	[46] 1
20–750	180 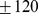	10–750	0.0019 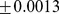	0.0025 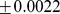	157 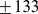	[47] 2
50–750	1080 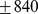	10–750	0.003 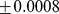	0.004 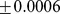	65 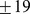	[47] 3
25–750	230 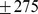	2.5–850	0.0014 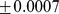	0.006 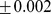	900	[48] 4
25–750	2332 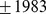	2.5–850	0.023 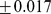	0.025 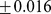	-	[48] 5
300–1050	300	150–900	0.0095	0.012	60	[49] 6
5–500	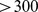	0–500	0.003	0.005	-	[50] 7
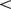 50–500	300–1200	25–500	0.0006	0.0029	720	[51] 8

Obs. 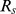: typical irradiance in shade and sunfleck (W m^−2^); Obs. 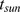: typical sunfleck duration (s); Exp. 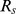: experimental range in irradiance (W m^−2^); Min. 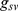: observed minimum stomatal conductance (m s^−2^); Max. 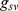: observed maximum stomatal conductance (m s^−1^); 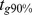: time to 90% of max. 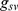 (s). Literature values of 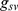 reported in units of mol m^−2^ s^−1^ were converted to m s^−1^ using Equation 19.

1 *Sorghum sp.*, lower leaves.

2 *Nothofagus cunninghamii*, coppice leaves.

3 *Nothofagus cunninghamii*, upper canopy leaves.

4 *Psychotria micrantha*, canopy gaps.

5 *Isertia haenkeana*, clearings.

6 *Triticum sp.*, Fig. 3.

7 *Pteridium aquilinum*.

8 *Acer rubrum*.

Note that the critical temperature of 49°C derived from the water submersion experiments is consistent with experimental results on the same species performed using heating in air [31]. It is remarkable that even for desert plants, extensive heat tissue damage commonly occurs close to the 50°C mark (up to 53°C, [32], [33]). This suggests that the function derived in this study from experimental data on black spruce may also be relevant for species in generally warmer habitats.

### Reference threshold for time to turgor loss after step change in irradiance

Assuming that the water supply rate from the xylem equals the steady-state leaf transpiration rate (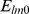, kg m^−2^ s^−1^) before the step change, we held this xylem supply rate constant and calculated the change in leaf water content (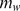, kg m^−2^) as the time integral of the dynamic transpiration rate (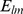, kg m^−2^ s^−1^) minus the initial steady-state transpiration rate. The time to turgor loss was taken as the time (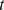, s) when the leaf water reservoir was depleted by 10%, i.e. when:
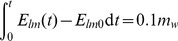
(32)The assumption that the xylem supply rate does not adjust within this time is likely to lead to an under-estimation of the critical time, whereas the assumption that the leaf is initially fully saturated and only loses turgor after 10% loss of its mass is likely to lead to an over-estimation of the critical time.

## Results

### Leaf temperature dynamics in a natural setting

**Figure 4:**
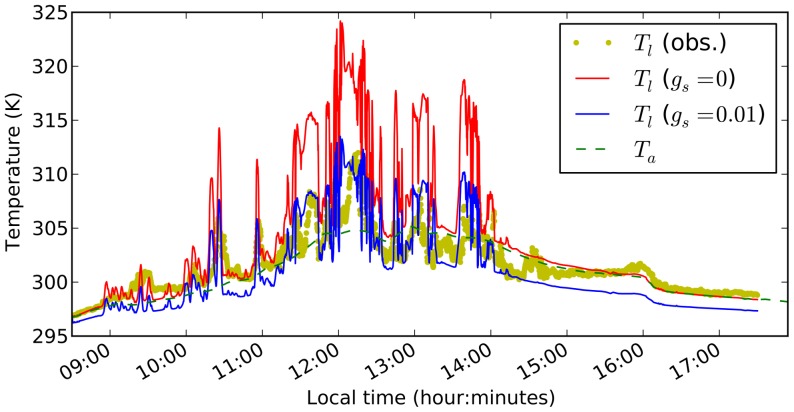
Observed and simulated leaf temperatures for an understorey plant in a tropical rain forest. Simulations are conducted for fully closed stomata (red) and a stomatal conductance of 0.01 m s^−1^ (blue). Observed leaf temperatures (yellow dots) and air temperatures (green dashed line) are taken from [19] and plotted against local time.

Using observations of diurnal variations in irradiance and air temperature in a tropical rainforest understorey [19], we simulated leaf temperature dynamics throughout the day considering a constant wind speed of 0.5 m s^−1^, and a constant atmospheric vapour pressure corresponding to 90% saturation at 8:30am, while varying irradiance and air temperature every 10 seconds. Using observed leaf temperature at 8:30am as an initial condition, we simulated three scenarios, one with fully closed stomata throughout the day (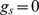 m s^−1^), one with a constant stomatal conductance of 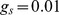 m s^−1^ and one with a non-limiting stomatal conductance (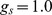 m s^−1^). The simulation using closed stomata tracked the observed leaf temperatures at the beginning and the end of the day, whereas the simulation with moderately open stomata tracked the observed leaf temperatures in the middle of the day (Fig. 4). The simulation with non-limiting stomatal conductance resulted in leaf temperatures well below observations throughout the day (data not shown). Note the large difference in simulated leaf temperatures in the middle of the day, depending on whether stomata are assumed open or closed.

**Figure 5:**
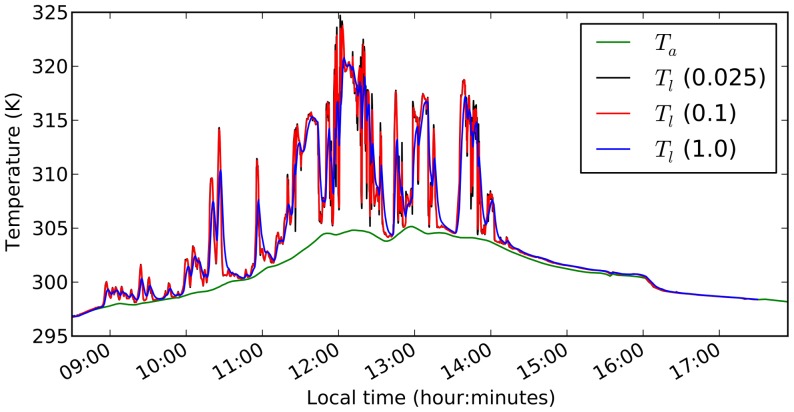
Simulated leaf temperatures in a rainforest understorey for closed stomata and different leaf water contents. Black: 0.025, red: 0.1 and blue: 1.0 kg m^−2^ leaf water content. The green line represents the observed air temperature [19], plotted against local time.

To assess how different leaf heat capacities could influence spikes in leaf temperature when stomata are closed, the same simulation were performed with different leaf water contents (0.025, 0.1 and 1.0 kg m^−2^). Results reveal that halving or doubling the estimated leaf water content at this site (0.05 kg m^−2^) did not have a large impact on simulated leaf temperature peaks (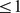 K), whereas a 20-fold increase in leaf water content to 1 kg m^−2^ could lead to a considerable reduction of simulated leaf temperature peaks by up to 5 K (Fig. 5). This suggests that the characteristic sunfleck durations are longer than the temperature time constants of the leaves at this site.

### Temperature dynamics for closed stomata

To understand the effect of a sudden increase in irradiance on a very hot day (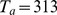 K or 40°C), we simulated the leaf temperature dynamics in response to a sudden increase in irradiance from 0 W m^−2^ (assuming that leaf temperature equals air temperature) to 400, 600 and 900 W m^−2^. We also plotted the critical temperature and exposure time relationship for heat damage in black spruce twigs as a reference, to assess in how far leaf heat capacity could delay heat damage. See Methods section 0 for details.

**Figure 6:**
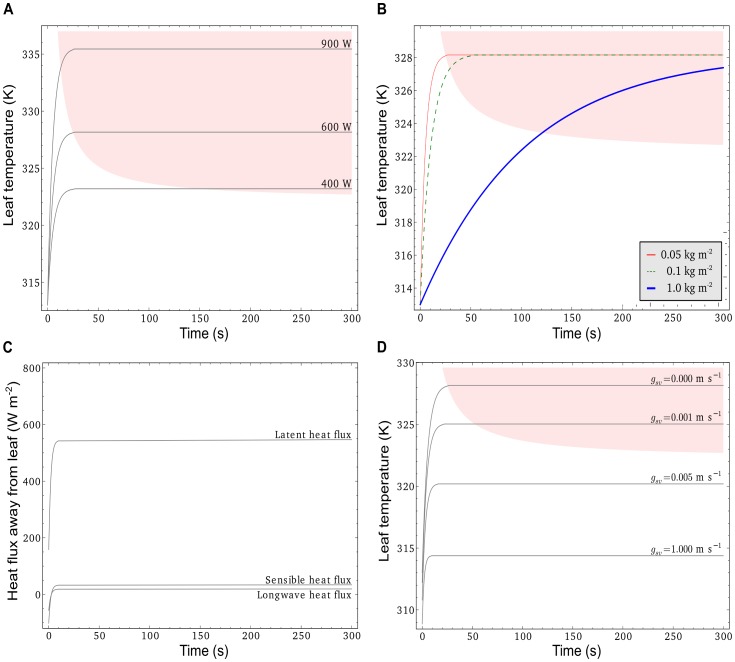
Leaf temperature and flux dynamics in response to sudden illumination. A: Temperature evolution of a non-transpiring leaf at different illumination intensities. B: Temperature evolutions of non-transpiring leaves with different water contents. C: Dynamics of latent, sensible and longwave heat flux from a leaf with non-limiting stomatal conductance (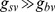). D: Temperature evolution of a transpiring leaf with different stomatal conductances (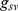). Common environmental conditions for all simulations: 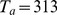 K, 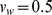 m s^−1^, 70% relative humidity, 0 W m^−2^ irradiance prior to arrival of sunfleck. Unless otherwise indicated, simulations are performed assuming a 5 cm wide leaf with 0.05 kg m^−2^ water content, exposed to 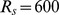 W m^2^ sunfleck irradiance. The shaded area represents critical combinations of leaf temperatures and exposure times that are expected to cause considerable heat damage. It is computed using the equation 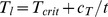, with 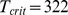 K and 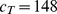 K s. This equation was derived from experimental data for black spruce needles (see Methods). In Panel (c), the calculated boundary layer conductance is 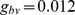 m s^−1^ and a stomatal conductance of 0.0029 m s^−1^, resulting in latent heat flux of 63 W m^−2^ prior to illumination and 248 W m^−2^ at steady state during the sunfleck, would be sufficient to keep leaf temperatures below 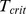.

The results suggest that steady-state temperatures are reached very fast (in less than a minute) for leaves with 0.05 kg m^−2^ water content and that non-transpiring leaves could heat up by up to 20 K in this time. For irradiances greater than 400 W m^−2^, sunflecks of less than two minutes duration could lead to heat damage (excursion into the shaded area in Fig. 6A). A 10-fold increase in leaf water content (from 0.1 kg/m2 to 1 kg/m2) could roughly quadruple the time to heat damage, from half a minute to two minutes for a sunfleck of 600 W m^−2^ intensity (Fig. 6B).

Increasing wind speeds (or decreasing leaf sizes) would have an increasing effect on sensible heat flux and a reducing effect on the steady-state temperatures but no effect on the time constants (data not shown).

### Temperature dynamics at constant stomatal conductance

When a leaf with open stomata is exposed to a sunfleck, the increase in leaf temperature may increase latent heat flux (Fig. 6C). However, evaporative cooling may concurrently suppress the rise in leaf temperature, leading to a lower steady-state leaf temperature than if stomata were closed. Fig. 6D illustrates the effect of evaporative cooling on the steady-state temperature of a leaf, for different stomatal conductances. Even low stomatal conductance (0.001 m s^−1^) could substantially reduce steady-state leaf temperature, and thus delay heat damage. Intermediate stomatal conductance values (0.005 m s^−1^) may reduce steady-state leaf temperature sufficiently to avoid risk of heat damage altogether. Note that the resulting latent heat flux at 600 W m^−2^ irradiance would be 126 and 319 W m^−2^ for the low and intermediate stomatal conductances respectively. This is equivalent to a transpiration of 2.2 and 5.6 mm respectively if integrated over 12 hours. In comparison, for fully open stomata, i.e. when stomatal conductance greatly exceeds boundary layer conductance (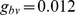 m s^−1^ in this case), the steady-state latent heat flux would be 547 W m^−2^, amounting to 9.6 mm of transpiration integrated over 12 hours (Fig. 6C). At a stomatal conductance of 0.0029 m s^−1^, steady-state leaf temperature would not exceed the critical temperature for heat damage of 322 K at 600 W m^−2^ illumination. The respective latent heat flux would be 248 W m^−2^, compared with 63 W m^−2^ for the same stomatal conductance in darkness (data not shown).

### Environmental conditions necessitating evaporative cooling

**Figure 7:**
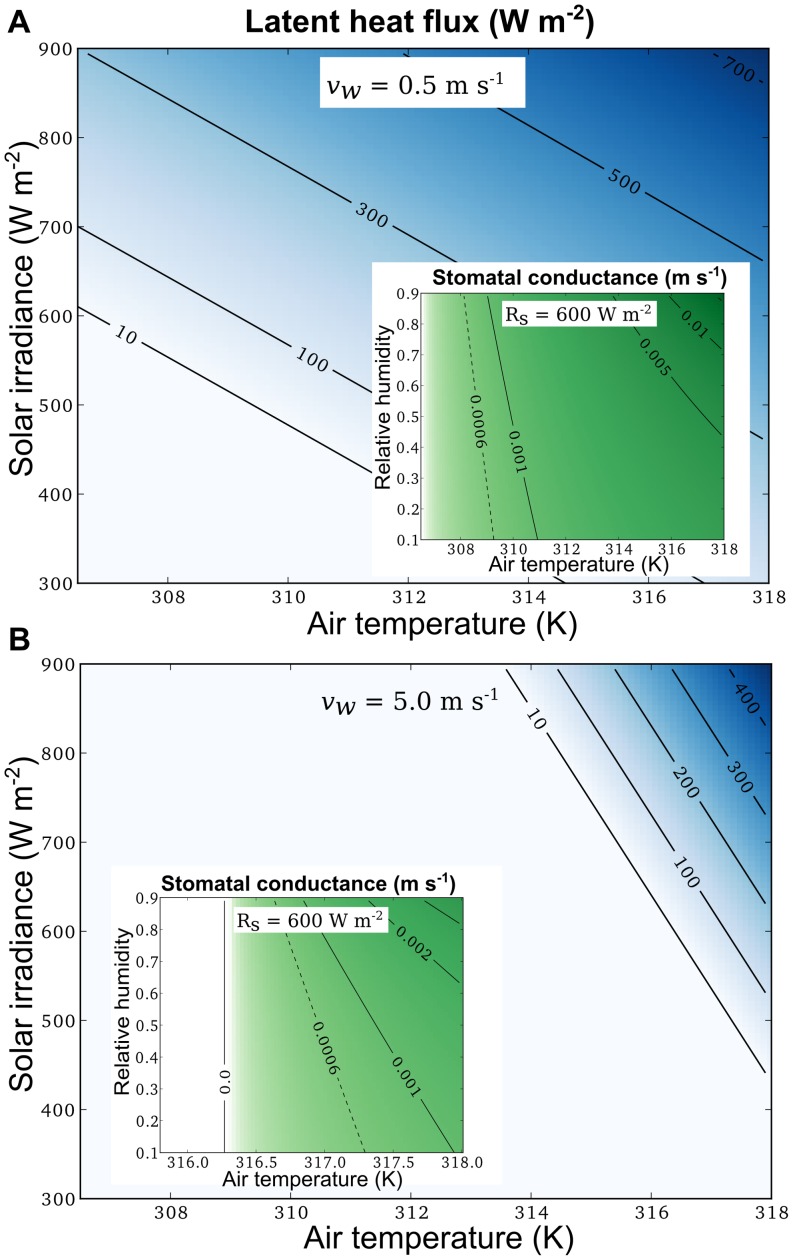
Rates of evaporative cooling and associated stomatal conductances to avoid heat damage. Contour lines in main panels represent rates of latent heat flux (W m^−2^) necessary to keep leaf temperatures at or below 322 K (49°C), for different combinations of air temperatures and solar irradiances (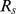). Panel A: assumed wind speed 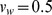 m s^−1^; Panel B: 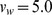 m s^−1^. Insets: stomatal conductances that would achieve the latent heat fluxes computed for 600 Wm^−2^ irradiance in main panels, for differrent relative humidities. Dashed contour lines mark the lowest stomatal conductance values observed in shaded leaves (Table 2).

Next, we wanted to know under what environmental conditions evaporative cooling is necessary for avoiding heat damage during very long sunflecks. Taking a leaf temperature of 322 K (49°C) as a critical temperature for heat damage (see Methods), we estimate the necessary cooling rate for different environmental conditions to maintain leaf temperatures below this critical value, considering a planar leaf with a characteristic length scale of 5 cm. Fig. 7A suggests that for low wind speeds (0.5 ms^−1^) and sunfleck intensity of less than 600 Wm^−2^, evaporative cooling would only be needed at air temperatures of more than 307 K (34°C). On the other hand, at air temperatures larger than 314 K (41°C), evaporative cooling is necessary for irradiance values as low as 300 W m^−2^ (Fig. 7A). Either increasing air temperature or relative humidity would require increasing values of stomatal conductance to achieve the necessary evaporative cooling.

For higher wind speeds (5.0 ms^−1^) cooling by sensible heat flux may become more vigorous and greatly reduce the need for evaporative cooling. This is expressed in Fig. 7B, where the need for evaporative cooling is limited to combinations of very high air temperatures and irradiance intensities. This in combination with a greatly increased leaf boundary layer conductance under high wind speeds also results in largely reduced stomatal conductances necessary to keep leaf temperatures below the critical value (inset in Fig. 7B).

### Critical arrival times to heat damage or turgor loss conditions

Leaf water content affects the slope of leaf temperature fluctuations, while stomatal conductance affects the amplitude. Hence, both affect the time to heat damage due to sudden illumination. However, increasing stomatal conductance also results in increasing additional water loss during illumination and increasing risk of turgor loss. The risk of turgor loss, on the other hand, can again be reduced by increasing leaf water content. It follows that leaf water content has a beneficial effect for both time to heat damage and time to turgor loss in response to a sudden increase in illumination. Here we ask the question about the relative importance of leaf water content for delaying heat damage or turgor loss.

**Figure 8:**
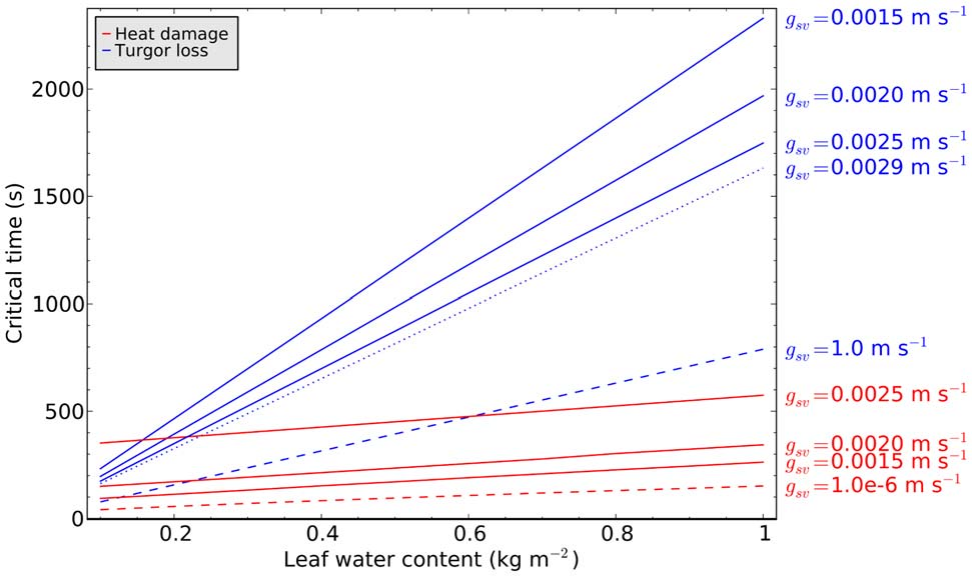
Critical exposure times to a sunfleck of 600 W m^−2^ light intensity for heat damage (red) or turgor loss (blue) as a function of initial leaf water content. Environmental conditions: 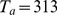 K, 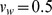 m s^−1^, 70% relative humidity, 100 W m^−2^ irradiance prior to arrival of light fleck. The steady-state transpiration rate at the pre-sunfleck light intensity was taken as a constant xylem water supply rate during the light fleck. Simulations were performed for different values of stomatal conductance, as indicated for each line on the right hand side. The dashed lines represent extreme cases of unlimited stomatal conductance (blue dashed) and negligible stomatal conductance (red dashed line). The blue dotted line represents the time to turgor loss if evaporative cooling is just sufficient to prevent heat damage altogether. In this case, latent heat flux rises from 90 W m^−2^ before sunfleck arrival to 248 W m^−2^ during the sunfleck.

Assuming initial steady-state between water loss by transpiration and leaf water supply by the xylem at 10 W m^−2^ irradiance, we abruptly increased irradiance to 600 W m^−2^ and considered the resulting increase in transpiration rate (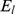) to be drawn from water stored in the leaf tissue. Note that the increase in latent heat flux (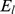) at constant conductance (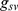) due to a step increase in radiation can be substantial, e.g. roughly 4-fold in 10 seconds for a leaf with 0.05 kg m^−2^ water content (Fig. 6C). Assuming that xylem water supply remains constant, the cumulative leaf water deficit was computed as the difference between the cumulative transpiration rate under the new radiation level and the transpiration rate at the initial level of 100 W m^−2^. For different values of constant stomatal conductance (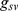), the time (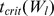) when water deficit reaches 10% of the intial leaf water content (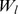) is plotted as a function of 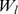 in Fig. 8. For the same values of 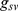, the critical time to heat damage (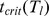) is also plotted as a function of initial leaf water content. Increasing 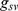 from 0.0015 to 0.0025 m s^−2^ could increase the time to heat damage very effectively, and when 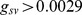 m s^−1^ heat damage would be avoided altogether in this case. At the same time, increasing 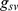 decreases the time to critical water loss, however much less effectively. Leaf water content has a much larger effect on the critical time to turgor loss than on the critical time to heat damage (different slopes of the respective red and blue lines). At 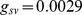 m s^−1^, which would be the necessary conductance for avoiding heat damage altogether, the resulting 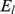 would be 66 W m^−2^ prior to the sunfleck and 248 W m^−2^ at steady state during sunfleck illumination, roughly half of the maximum possible 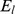 of 547 W m^−2^ at non-limiting 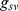.

## Discussion

### Simulated and observed leaf temperatures

Rapid fluctuations in leaf-incident irradiance, e.g. due to moving sunflecks within a canopy, can result in large and rapid increase in leaf temperatures [19] to critical levels (see also Fig. 2). The leaf energy balance model presented here was capable of reproducing the observed diurnal variation in leaf temperature in the canopy of a tropical rainforest, when stomatal conductance was set to 0 early and late in the day, and a moderate value in the middle of the day (Fig. 4). Some deviations between simulated and observed leaf temperatures were expected, as stomatal conductance and wind velocities were not measured, so the simulations were run with a constant wind velocity (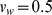 m s^−1^) and constant stomatal conductance. Correspondence between the red line (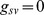) and observations in the morning suggests that stomata are closed in the morning and confirms the correct representation of sensible heat flux in the model. Correspondence between the blue line (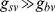) and observations in the middle of the day suggests that stomatal conductance did not vary much between sunflecks. It is interesting to note that on some occasions, low leaf temperatures are best captured by the red line, while high leaf temperatures during sunflecks are better captured by the blue line (e.g. 10–11am in Fig. 4). This could suggest that stomata open during sunflecks and close in between. In the middle of the day (between 11:30am and 2:30pm) the red line stays well above the observed leaf temperatures, which could suggest that stomata stay open all the time, or that wind velocities are higher than the assumed 0.5 m s^−1^. The effect of wind fluctuations could also be responsible for the leaf temperature jump around 2:30pm, when the observed leaf temperatures alternate between the red and the blue lines. The leaf temperature jump around 4pm cannot be explained based on the available data, as solar irradiance is near 0 and the jump exceeds air temperature.

### Protection from over-heating

Simulated leaf temperatures for closed stomata have reached values of up to 325 K (52°C) in the middle of the day (Fig. 4). Given observed durations of the sunflecks [4], the leaf intrinsic heat capacity is incapable of significantly reducing leaf temperature peaks. Reducing the leaf water content by 50% would not significantly increase peak leaf temperatures, whereas a 20-fold increase in leaf water content could reduce peak leaf temperatures by 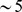 K (Fig. 5).

Theoretical modelling results confirm that the increase in leaf temperature as a result of a step increase in irradiance on a hot summer day can be rapid enough to reach potentially damaging leaf temperatures even for relatively short-lived sunflecks (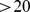 K increase in half a minute for non-transpiring leaves, Fig. 6A). The increase in heat capacity related to an increase in leaf water content from 0.05 to 0.1 kg m^−2^ would only delay critical leaf temperatures by a few seconds (Fig. 6B). Only leaf water contents of 1 kg m^−2^ and more would slow down the temperature rise sufficiently to delay heat damage by two minutes or more in our example (Fig. 6B). However, such thick leaves are not common in closed canopies where rapid variations in irradiance are most pronounced. This suggests that the leaf heat capacity is not commonly used by plants to mitigate increases in leaf temperature due to sun flecks.

Thick and watery leaves are commonly found in deserts, among succulent plants with crassulacean acid metabolism (CAM), which keep their stomata closed during the day to conserve water. Desert plants are usually fully exposed to sunlight and rely on cooling by mainly radiative and sensible heat flux. In a recent study, Leigh et al. [34] investigated the protective role of leaf heat capacity against thermal damage in four desert plant species during short periods with low wind speeds. They simulated leaf temperatures for 0.2 mm thick leaves in comparison to realistic leaves of 0.4–0.6 mm thickness and found that with thinner leaves, two out of the four species could become heat damaged. However, the authors did not consider exposure times to high leaf temperatures as a damaging factor while the differences in maximum leaf temperatures between thin and thick leaves were less than 0.5 K, consistent with our results.

Our analysis suggests that transpiration-induced cooling is much more effective in avoidance of high leaf temperatures than capacitive delays following exposure to sunflecks. However, for this mechanism to be effective, leaves must either keep their stomata open even in the shade, or be able to open stomata rapidly following sunfleck exposure. If stomata are already open, the sunfleck-induced increase in leaf temperature can result in substantial increase in leaf latent heat flux, which in return suppresses overall leaf temperature increase (Figs. 6C and 6D). It is primarily the reduction in steady-state leaf temperature and not the time to maximum temperature, that determines the effectiveness of transpirative cooling on the critical time to heat damage (Fig. 6D). In contrast, leaf water content does not affect the steady-state temperature during sunfleck exposure, but the rate of temperature rise and therefore arrival time and duration of exposure to damaging temperatures. Even if steady-state temperatures are reached immediately, e.g. in a leaf with negligible heat capacity, heat damage does not happen immediately, but is a function of the exposure time [30]. Therefore, the red lines in Fig. 8 intersect the ordinate at a level determined by the steady-state leaf temperature. Increasing leaf water content (i.e. heat capacity) increases the time until a critical leaf temperature (322 K in our examples) is reached in a roughly linear fashion (see Fig. 6B). This time should be added to critical exposure duration that is largely determined by steady-state temperature. For situations where steady-state temperature greatly exceeds the critical leaf temperature, the capacitive delay time to critical temperature becomes a significant factor in the onset of heat damage. For lower steady-state temperature, the thermal capacity becomes less significant, as the critical exposure duration is much longer than the time to critical leaf temperature.

### Protection from hydraulic failure

As shown previously, a rapid increase in transpiration rate in response to a sunfleck is an effective protection mechanism against over-heating, but it also exposes plant leaves to the risks of turgor loss and/or cavitation [9]. Zimmermann [35] proposed that the hydraulic system of trees is segmented to prevent cavitation in trunks by imposing preferred cavitation in leaf petioles and roots before pressure drops propagate into the trunks. Zwieniecki et al. [36] found that the hydraulic conductance in petioles declines in response to a drop in leaf water potential, but recovers quickly when leaf water potential is restored, as long as the metabolism of living cells is not inhibited. This was regarded as an indication of active refilling of emobilised vessels, which requires the expenditure of energy by living tissues [36]. The proximity of living and photosynthesising tissues in leaf petioles may allow easier recovery from cavitation than in the trunk xylem, which could implicate petioles as “safety valves” [18] for accommodating cavitation before effects of rapid pressure drops can propagate into the trunk xylem. The hydraulic conductivity in the petiole determines the pressure jump between the leaf and the trunk xylem for a given flux rate. Thus, a decrease in petiole conductivity with decreasing leaf water potential could have a stabilising effect on the xylem pressure, as it would make the difference between leaf and xylem water potential increase (at a constant flux rate). Note that the role of a “safety valve” could equally be performed by the leaf tissue itself, if the shrinkage of parenchyma tissue at the end of xylem elements due to tissue water loss resulted in a reduction in hydraulic conductivity between xylem and parenchyma tissue. Based on rehydration experiments, Zwieniecki et al. [37] proposed different levels of leaf compartmentalisation that determine the connectivity of different leaf tissues with the xylem: (1) xylem is separated from leaf tissues by a low conductivity barrier, (2) xylem is linked to epidermis but mesophyll is separated by low conductivity barrier and (3) all leaf tissues are linked to the xylem. The low conductivity barriers are zones where the largest pressure drops occur during steady flow, so the three different scenarios determine which tissues are relatively depleted of water before turgor-induced stomatal closure. For case (1), the pressure drop would occur between the leaf xylem and all other tissues, i.e. stomata would close autonomously when the entire leaf tissue reaches a critical water depletion and potential, whereas the leaf xylem potential would remain relatively unchanged. In case (2), autonomous stomatal closure would be expected when water depletion in the epidermis becomes critical, while the mesophyll can maintain higher water potential. In this case, the leaf xylem potential would be expected to decline together with the water potential of the epidermis. In case (3), like in case (1), all leaf tissues would reach a critical water depletion and potential before autonomous stomatal closure, but in this case, the leaf xylem potential would also decline.

We investigated the role of hydraulic capacitance determined by leaf water content as an autonomous reservoir supplying the increased transpiration rate without affecting xylem status, i.e. considering unperturbed xylem water supply. Results show that even for fully open stomata, the increase in transpiration rate induced by a 600 W m^−2^ sunfleck could be accommodated for several minutes in leaves with water content 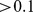 kg m^−2^ (blue dashed line in Fig. 8). This is in contrast with Zwieniecki et al. [37], who assumed that the leaf mesophyll would only support transpiration for tens of seconds. The critical time to turgor loss at constant xylem water supply is a linear function of the leaf water content (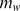), with a slope that scales with the inverse of transpiration rate (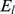). Therefore, the lines in Fig. 8 become steeper with decreasing stomatal conductance (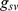).

Our analysis relates to leaf compartmentalisation scenarios (1) or (3) in the above description, as we assumed that all turgid tissues in the leaf can contribute up to 10% of their water content to the transpiration stream. The analysis is not applicable to leaves of design (2), where stomatal closure is expected already when the leaf epidermis becomes water-depleted. Such leaves either have to be coupled to a very efficient water supply system that responds to pressure drops by increased supply rate, or avoid the combination of sunflecks and high air temperatures. Note that the water content of the leaves described in Figure 4 was only near 0.05 kg m^−2^, and they were exposed to sunflecks of more than 5 minutes duration without signs of stomatal closure in the leaf temperature data (Fig. 4). This suggests that these plants do have an efficient xylem water supply that can adjust to fluctuating leaf water demand within minutes (the time scale of leaf water depletion according to our analysis).

### Implications for stomatal adjustments

The numerical experiments revealed that keeping stomata partly open in shaded leaves provides effective protection from over-heating when a leaf is suddenly exposed to a sunfleck (Figs. 6D and 8). For a step exposure to 600 W m^−2^ irradiance intensity and conditions as in Fig. 8, the stomatal conductance (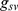) should be roughly one quarter the leaf boundary layer conductance (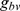) to provide effective protection. With this stomatal conductance in the shade, water would be lost at a rate of 90 W m^−2^, which is equivalent to 16% of the maximum possible transpiration rate at 600 W m^−2^ irradiance (547 W m^−2^, Fig. 6C). Considering the low photosynthetic rates in the shade, such transpiration rate (16% of the maximum possible rate) represents considerable water loss, particularly if shaded leaves are exposed to only a few short sunflecks in a day. Thus, in a water-limited environment, it may be beneficial for plants to maintain closed stomata in the shade and only open during sunfleck exposure. For conditions as in Fig. 8, stomata must start opening within a minute and reach values of roughly 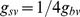 within 5 minutes to avoid heat damage. Considering typical stomatal response times of 2–60 minutes [2], our analysis implies that keeping stomata open is critical for avoidance of sunfleck-induced thermal damage on hot days with little wind. We found this confirmed in the observed leaf temperature data in Fig. 4, which was consistent with our simulations assuming open stomata even during low light periods in the middle of the day.

Using the red dashed line in Fig. 8 as a reference, we searched the literature for observations of 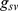 in sun and shade in environments with sunflecks of 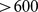 W m^−2^ intensity and 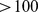 s duration. The results are summarised in Table 2. Note that the minimum stomatal conductances reported in Table 2 may under-estimate the stomatal conductance of a leaf just before it is hit by a strong sunfleck, as in many environments strong sunflecks are preceded by a series of weaker sunflecks in the morning, which already induce stomatal opening [38]. Keeping this in mind, it is remarkable that the minimum conductances observed in the shade are generally high enough to avoid critical leaf temperatures at air temperatures of more than 309 K (36°C), as implied by the dashed lines in Fig. 7 B. In the extensive data compilation by Vico et al. [2], initial values of stomatal conductance range between 0.00002 and 0.075 m s^−2^, with a median value of 0.0035 m s^−2^. Note that 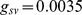 m s^−2^ is close to the conductance necessary to completely avoid the danger of heat damage under the conditions simulated in Fig. 8.

Unfortunately, the growth conditions in the different studies were not documented in sufficient detail with respect to sunfleck intensities and durations as well as wind velocities, air temperatures and humidity to correlate observed shade conductances with those necessary to survive naturally occurring sunflecks. Furthermore, the measurements were usually performed under conditions that did not pose a risk of over-heating to the leaves, as air temperatures were not very high, while air flow in the leaf cuvettes was relatively high. To shed more light onto the links between avoidance of heat damage and stomatal adjustments, more experimental research is needed under potentially temperature-stressed conditions, i.e. high air temperatures and low wind speeds.

To date, open stomata in the shade have been generally regarded as a measure to alleviate stomatal limitations to CO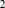 uptake in the early periods of sunflecks, given the restrictions on stomatal opening rates (see e.g. [2], [39]). Our modelling results suggest that on hot days with temperatures above 308 K (35°C), open stomata in the shade may have another, potentially much more vital role, namely protection from high leaf temperatures during sunflecks. The former function is expected to become relatively more important for short sunflecks (e.g. 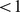 min), where closed stomata would not result in overheating anyway, but in very low total sunfleck light use, whereas the latter function is expected to become relatively more important for sunflecks that are long enough to lead to critical leaf temperatures in leaves with closed stomata (e.g. 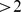 min). The examplary sunfleck durations mentioned here are deduced from the red dashed line in Fig. 8, but note that these critical times would vary with different levels of leaf heat tolerance, different wind velocities and air temperatures as well as different sunfleck light intensities. We do not imply that leaf temperature control is the major driver for stomatal adjustments, as the need to achieve sufficient CO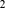 uptake during sunflecks may result in sufficiently high stomatal conductances to avoid the danger of heat damage anyway. However, our simulations suggest that water stress on hot summer days may not only have a negative impact on the leaf carbon balance and lead to starvation, but in fact is likely to have a much more immediate effect by leading to heat damage.

### Heat damage under water stress

As discussed above, the potential for a single sunfleck of sufficient intensity and duration to damage non-transpiring leaves (Fig. 6A), combined with the relatively slow stomatal response suggest that open stomata in shaded canopies should be relatively common. On the other hand, under limited soil water supply, keeping stomata open throughout the day may not be feasible and thus limit the ability to simultaneously avoid heat damage and hydraulic failure.

A potential adaptation is increased heat tolerance in response to drought. In fact, drought preconditioning has been found to improve heat resistance in a range of plants (see [40] and references therein), suggesting that stronger limitation in evaporative cooling necessitates greater heat tolerance. In a review of mechanisms of drought damage to trees, Hartmann [41] quotes evidence that trees grown in higher temperatures had a higher mortality in response to drought than plants grown under normal temperatures. Conventional explanations attribute this to higher respiration rates under elevated temperatures and thus higher risk of carbon starvation for trees that must close stomata under drought. So far, these explanations have not yet been supported by evidence [42]. Hartmann [41] recommended analysing alternative hypotheses, such as symplastic failure or inhibition of the redistribution of assimilates, both due to low tissue water potentials.

In view of our study, we would propose to also look at heat damage as a result of reduced evaporative cooling under drought. This might explain increased mortality under elevated temperatures, whereas tissue water potential-related mechanisms alone cannot easily explain these observations. More evidence supporting our heat damage hypothesis was provided by Warren et al. [43], who found that a heat wave combined with drought led to increased leaf senescence under elevated CO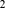 treatments compared to ambient CO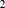 concentrations. If elevated CO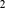 leads to lower stomatal conductances per leaf area or increased carbon gain (or both), then it should be expected to alleviate starvation issues and/or increase the heat damage risk. Warren et al. [43] documented a strong decrease in canopy conductance under elevated CO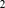 and no increased carbon gain. The increased leaf senescence under elevated CO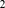 during the drought was attributed to stomatal closure, increased leaf temperatures and reduced carbon gain [43]. Our study suggests that the increased leaf senescence and reduced carbon gain may also be explained by direct heat damage, particularly as it occurred during the “hottest time of the year, as 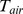 reached 38°C” [43].

Other protective measures from heat damage in times of inadequate water supply could include reduced absorption of sunlight due to wilting [9], vertical leaf inclination or high leaf reflectivity, and enhanced sensible heat flux by very small leaves.

Okajima et al. [44] documented a decreasing trend of leaf size with increasing mean annual temperatures within the same species, and argued that this correlation may be a result of optimising steady-state leaf temperatures for maximising photosynthesis. For species with an increasing lack of occurrences of large leaves at higher mean annual temperatures, but no lack of small leaves at low temperatures (at least half of the examples presented in [44]), we would argue that avoidance of heat damage may be a better explanation of the pattern. Only for species with a lack of small leaves at low temperatures is the photosynthesis-based explanation more plausible.

### Conclusions

Our analysis suggests that leaf water content has a dual protective role in leaves exposed to short but intense sunflecks. On the one hand, it can delay the onset of heat damage due to its effect on the leaf heat capacity, and on the other hand it provides a buffer for fluctuations in evaporative losses and thereby delays turgor loss when a leaf with open stomata is exposed to a sudden increase in illumination. Our analysis further suggests that keeping stomata open before a sunfleck arrives is likely a vital strategy to avoid heat damage during the sunfleck on a hot day. This finding is consistent with a wide range of studies where initial stomatal conductances prior to the arrival of sunflecks were documented. This may have implications for daily water use efficiencies, but also suggests that drought conditions may result in heat damage to leaf tissues on hot days offering an alternative explanation for the damaging effect of simultaneous drought and heat waves on vegetation. In this context, clouds or aerosols in the atmosphere should not only allow higher photosynthesis rates in deeper canopies due to more diffuse light [45] but also reduce the intensity of sunflecks and hence allow an overall higher water use efficiency and lower the risk of heat damage due to sunflecks.

In conclusion, we can answer the questions formulated in the introduction as follows:

- Do natural fluctuations in leaf irradiance necessitate stomatal regulation to avoid heat damage or hydraulic failure?

On hot summer days, a sunfleck could cause heat damage to a non-transpiring leaf within a minute, whereas moderate stomatal conductance can result in sufficient evaporative cooling to avoid heat damage under most realistic conditions. Since observed time scales of stomatal adjustments are generally longer than a minute, stomata need to be already partly open when a sunfleck arrives, in order to allow for autonomous evaporative cooling as the leaf heats up. Common variations in leaf water content are sufficient to supply 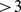 minutes worth of transpiration without propagating a pressure drop into the xylem, even for large stomatal conductances (Fig. 8). Since the combination of leaf water capacity and hydraulic xylem efficiency has to be able to support sufficient evaporative cooling on hot days, it is unlikely that stomatal down-regulation of evaporation would become necessary during a sunfleck.

- What is the role of leaf heat and water capacities in negotiating the trade-off between cavitation and over-heating?

In typical canopy leaves, leaf heat capacity contributes only little to extending the time to heat damage during a sunfleck. For a variation in thermal capacitance by one order of magnitude, the simulated time to heat damage only increased by 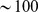 s (Figs. 6B and 8). In contrast, the same range of variation in leaf water capacity extends the time to critical dehydration during a sunfleck roughly 10-fold, e.g. from 200 to 2000 s (Fig. 8).
